# Chikungunya Fever, Andaman and Nicobar Islands, India

**DOI:** 10.3201/eid1308.070193

**Published:** 2007-08

**Authors:** Sathya P. Manimunda, Shiv S. Singh, Attayoor P. Sugunan, Omkar Singh, Subarna Roy, Ananganallur N. Shriram, A. P. Bharadwaj, Wajid A. Shah, Paluru Vijayachari

**Affiliations:** * Regional Medical Research Centre, Andaman and Nicobar Islands, India; † GB Pant General Hospital, Port Blair, Andaman and Nicobar Islands, India

**Keywords:** Chikungunya fever, India, Andaman and Nicobar Islands, letter

**To the Editor:** The outbreak of chikungunya fever that started in the Indian Ocean Islands in early 2005 (*1*) spread through adjoining islands and appeared in peninsular India by late 2005 (*2*). It was first noticed in the southern state of Andhra Pradesh in February 2006; it spread to Tamil Nadu in April 2006 and to Karnataka and Kerala in May. The western state of Gujarat also reported cases in April, but no cases were reported in May and June. The disease again reappeared in July and reached a peak in August. Later it affected the central Indian states of Maharashtra and Madhya Pradesh. In most states, the outbreak declined by October 2006 (*3*,*4*).

Andaman and Nicobar Islands, a union territory of India, is an archipelago of >500 islands and islets situated in the Bay of Bengal, 1,200 km from peninsular India. People are constantly moving between mainland India and these islands. Chikungunya fever has previously not been reported from these islands.

During July and August 2006, medical professionals noticed an increase in the number of cases of febrile illness in Port Blair, the headquarters of the union territory and the only urban area in the islands. The total number of patients with fever who visited the 5 urban health centers (UHC) in the town went up from the baseline of 300–450 per day to 550–900 per day in July and August 2006. Most of the patients had associated joint pain. In view of the clinical features suggestive of chikungunya fever, the ongoing epidemic on mainland India, and the widespread presence of the vector, *Aedes aegypti,* within the urban area of Port Blair (*5*), chikungunya fever was suspected. To confirm this hypothesis, 17 persons who fulfilled the case definition of having an acute febrile illness associated with severe pain in multiple joints were selected from among the initial patients who went to the UHCs and the referral hospital in Port Blair. Among these study participants, 15 were adults and 2 were adolescents 15 years of age; 6 were female and 11 male. Four adults had febrile illness associated with joint pain; in these patients, weakness of all 4 limbs developed 3–15 days after onset of illness. All of the 4 patients with weakness had areflexic quadriplegia; 1 required ventilatory support. The patients with areflexic quadriplegia were treated with injections of methylprednisolone; all recovered within a week.

Blood samples were collected from these study participants. Serum samples were separated and sent to the National Institute of Virology, Pune, for detection of anti–chikungunya virus (CHIKV) immunoglobulin M (IgM) antibodies. Samples were collected from 12 patients >4 days after the onset of symptoms. In the remaining patients, the interval between onset of symptoms and collection of blood samples was <4 days. Of the 17 study participants, 13 were positive for anti-CHIKV IgM antibodies. Three of 4 samples that were negative for IgM antibodies to CHIKV were collected <3 days after the onset of symptoms. Among these, 2 samples were subjected to reverse transcriptase–PCR by using the primers CHIKV/E1S (5′-TAC CCA TTC ATG TGG GGC-3′) and CHIKV/E1C (5′-GCC TTT GTA CAC CAC GAT T-3′), as described by Hasebe et al. (*6*); both were positive for CHIKV RNA. All these samples were tested for dengue IgM antibodies by using SD Bioline Dengue IgM Rapid Test (Standard Diagnostics Inc., Kyonggi-Do, South Korea), which uses a mixture of dengue recombinant envelop proteins and can detect all of the 4 dengue serotypes. None of the samples tested positive for dengue antibodies. Hence, CHIKV infection was confirmed in 15 of 17 patients.

India experienced the first confirmed outbreak of chikungunya fever in 1963–1964 in Kolkata (*7*) and in 1965 in Chennai. The last epidemic in India was reported from Barsi in the state of Maharastra in 1973 (*8*). However, during these outbreaks, Andaman and Nicobar Islands were not affected. Outbreaks of dengue fever and chikungunya fever are known to occur simultaneously, as has happened in several parts of India. However, during the current outbreak in Andaman Islands, dengue infection was not detected. (Dengue has never been reported in the islands.) As chikungunya fever is known for its mysterious pattern of dramatic outbreaks interspersed by periods of prolonged absence, the introduction of this virus to an unexposed population has great public health importance.

This outbreak could be a warning about preparedness for health authorities not only in these islands but also in other areas where chikungunya fever has not occurred previously. With the extent of human travel to and from areas with active chikungunya virus transmission, many areas where the disease has not previously been reported could be at risk. As an outbreak response, the Regional Medical Research Centre and Directorate of Health Services, Andaman and Nicobar Administration, has undertaken a comprehensive community-based survey to assess the impact of chikungunya fever and *Aede*s infestation levels. We are stepping up our applied field research to prevent future outbreaks of chikungunya fever, as well as dengue fever.
